# From Needle to Sachet: Fosfomycin Versus Amikacin for Cystoscopy Prophylaxis

**DOI:** 10.7759/cureus.102755

**Published:** 2026-01-31

**Authors:** Uvaisullah Quadir, Sajad A Para, Sajad A Malik, Arif Hamid, Abdul R Khawaja, Saqib Mehdi, Syed Shakeeb Arsalan, Aamir B Raina, Gokul Kannan, Omar Mukhtar Bhat

**Affiliations:** 1 Department of Urology, Sher-i-Kashmir Institute of Medical Sciences, Srinagar, IND

**Keywords:** amikacin, antibiotic prophylaxis, cystoscopy, fosfomycin, lower urinary tract infection

## Abstract

Background: Antibiotic prophylaxis is commonly administered before outpatient cystoscopic procedures (CPE). This study compared the efficacy and safety of parenteral amikacin and oral fosfomycin used as prophylactic agents before outpatient cystoscopy.

Methods: This retrospective comparative observational study included adult patients undergoing outpatient CPE at a tertiary care center between October 2024 and November 2025. Patients received either a single intravenous dose of amikacin (15 mg/kg) or a single oral dose of fosfomycin trometamol (3 g) before the procedure, according to institutional practice. The primary outcome was the incidence of symptomatic urinary tract infection (UTI) within 14 days following CPE. Secondary outcomes included culture-positive UTI and adverse drug reactions (ADRs). Categorical variables were compared using the chi-square or Fisher’s exact test, and continuous variables were compared using the independent t-test.

Results: A total of 2914 patients were included, of whom 2083 received amikacin, and 831 received fosfomycin. Symptomatic UTI occurred in 6.7% of patients in the amikacin group and 8.2% in the fosfomycin group (p = 0.18). Culture-positive UTI rates were comparable (2.1% vs 2.0%, p = 0.91). Acute kidney injury was significantly more frequent with amikacin (2.6% vs 0.1%, p < 0.001), while gastrointestinal adverse effects were more common with fosfomycin (8.3% vs 2.3%, p < 0.001). Injection-related reactions occurred only in the amikacin group.

Conclusions: Fosfomycin demonstrated efficacy comparable to amikacin for preventing post-cystoscopy UTI, with a more favorable safety profile. Oral fosfomycin represents a safe and effective alternative to aminoglycosides for prophylaxis before outpatient cystoscopic procedures, particularly in patients at risk for aminoglycoside-related toxicity.

## Introduction

Cystoscopic procedures (CPE) represent one of the most commonly performed interventions in urological practice, serving both diagnostic and therapeutic purposes. These include surveillance for non-muscle-invasive bladder cancer, evaluation of hematuria and lower urinary tract symptoms, urethral assessment, and removal of ureteral stents following endourological procedures. Although CPE is generally regarded as a low-risk procedure, post-procedural urinary tract infection (UTI) remains a recognized complication that can lead to patient morbidity, unplanned healthcare visits, and additional antibiotic exposure [1].

The reported incidence of post-cystoscopy UTI varies widely and is influenced by patient-related risk factors such as diabetes mellitus, prior history of UTI, indwelling urinary catheterization, advanced age, immunosuppression, and underlying lower urinary tract pathology. Procedural factors, including duration of instrumentation and mucosal trauma, may further increase infection risk. Consequently, antibiotic prophylaxis continues to be widely practiced in selected patients, despite growing concerns regarding antimicrobial resistance and the need for antibiotic stewardship [2].

Guidelines from the European Association of Urology (EAU) and the American Urological Association (AUA) advocate a risk-stratified approach to antibiotic prophylaxis for cystoscopic procedures. Routine prophylaxis is generally not recommended for low-risk diagnostic cystoscopy, while selective prophylaxis may be considered in patients with identifiable risk factors for infection. However, specific recommendations regarding the choice of antibiotic agent are limited, resulting in substantial variability across institutions and regions [3,4].

Aminoglycosides such as amikacin have traditionally been used as single-dose prophylactic agents because of their potent activity against Gram-negative uropathogens and rapid bactericidal action. Nonetheless, concerns regarding nephrotoxicity, ototoxicity, and the requirement for parenteral administration limit their desirability, particularly in the outpatient setting. Even single-dose exposure may be relevant in susceptible patients with borderline renal function or multiple comorbidities [5].

Fosfomycin trometamol has emerged as a potential alternative for urological prophylaxis. Administered orally as a single dose, fosfomycin achieves high urinary concentrations and retains activity against common uropathogens, including extended-spectrum beta-lactamase (ESBL)-producing organisms. Its favorable safety profile and ease of administration make it attractive for outpatient use. However, higher direct drug cost and limited large-scale real-world comparative data have constrained its widespread adoption [6,7].

In this context, real-world comparative studies are needed to guide antibiotic selection in alignment with contemporary guidelines. The present study compares amikacin and fosfomycin as prophylactic antibiotics before outpatient CPE, focusing on post-procedural UTI rates and adverse drug reactions (ADRs), with the aim of informing guideline-consistent and patient-centered prophylactic strategies [8,9]. Hence, the primary objective was to compare post-procedural symptomatic UTI rates between amikacin and fosfomycin, while secondary objectives included comparison of symptomatic and culture-positive UTI, along with ADRs.

## Materials and methods

Study design and population

This retrospective comparative observational study was conducted at the Department of Urology, Sher-i-Kashmir Institute of Medical Sciences (SKIMS). All adult patients aged 18 years or older who underwent outpatient cystoscopic procedures (CPE) between October 2024 and November 2025 were evaluated.

Inclusion and exclusion criteria

Patients were included if they were aged ≥18 years, underwent outpatient CPE, and had either a sterile urine culture or asymptomatic bacteriuria before the procedure. Patients with active UTI at the time of CPE, known hypersensitivity to amikacin or fosfomycin, immunosuppressed status, or incomplete medical records were excluded. Patients with asymptomatic bacteriuria were not considered to have active infection. Antibiotic prophylaxis was administered selectively as part of routine institutional practice.

Prophylactic antibiotic regimen

Antibiotic prophylaxis was administered according to institutional practice. Patients received one of the following regimens. The amikacin group received a single intravenous dose of amikacin (15 mg/kg) administered 30-60 minutes before CPE. The fosfomycin group received a single oral dose of fosfomycin trometamol (3 g) administered two to three hours before CPE. No routine postoperative antibiotics were prescribed unless clinically indicated.

Data collection

Data were collected retrospectively from hospital medical records and procedure logs. Patients were followed retrospectively for 14 days, going through documents of outpatient visits and review of hospital records for urinary symptoms, culture results, and ADRs. Variables recorded included age, sex, comorbidities including diabetes mellitus, presence of a per-urethral catheter at the time of CPE, history of prior UTI, indication for CPE, prophylactic antibiotic administered, post-procedural urinary symptoms, urine culture results, and ADRs.

Outcome definitions and tools

The primary outcome was the incidence of symptomatic UTI within 14 days following CPE. Symptomatic UTI was defined as the presence of urinary symptoms, including dysuria, urinary frequency or urgency, suprapubic pain, or fever requiring antibiotic therapy [10]. Culture-positive UTI was defined as the growth of a single uropathogen at a concentration of ≥105 colony-forming units per milliliter on urine culture [11]. Secondary outcomes included ADRs. Acute kidney injury was defined according to the KDIGO (Kidney Disease: Improving Global Outcomes) criteria as an increase in serum creatinine to ≥0.3 mg/dL from baseline [12]. Gastrointestinal adverse effects included nausea, vomiting, diarrhea, and abdominal discomfort. Injection-related reactions, allergic reactions, ototoxicity based on symptoms, and serious adverse events were also assessed. ADRs were identified through clinical documentation and laboratory data during follow-up.

Statistical analysis

Categorical variables were expressed as frequencies and percentages, while continuous variables were expressed as mean ± standard deviation. Comparisons between groups were performed using the chi-square test or Fisher’s exact test for categorical variables and the independent Student’s t-test for continuous variables. A p-value of <0.05 was considered statistically significant.

Ethical considerations

The study protocol was approved by the Institutional Ethics Committee of SKIMS. As this was a retrospective observational study, informed consent was waived. Patient confidentiality was strictly maintained, and the study involved no deviation from standard clinical care.

## Results

During the study period from October 2024 to November 2025, a total of 2914 patients underwent outpatient cystoscopic procedures (CPE) at the Urology lab, Department of Urology, SKIMS. Among these, 2083 patients (71.5%) received amikacin as prophylactic antibiotic, while 831 patients (28.5%) received fosfomycin.

In the fosfomycin group, 588 patients (70.8%) were male, and 243 patients (29.2%) were female, whereas in the amikacin group, 1641 patients (78.8%) were male, and 442 patients (21.2%) were female. The mean age of patients was 47 ± 12 years in the fosfomycin group and 51 ± 14 years in the amikacin group.

Baseline clinical characteristics

Baseline clinical characteristics were broadly comparable between the two groups. Diabetes mellitus was present in 97 patients (11.7%) in the fosfomycin group and 214 patients (10.3%) in the amikacin group. A per-urethral catheter (PUC) at the time of CPE was documented in 39 patients (4.7%) in the fosfomycin group and 88 patients (4.2%) in the amikacin group. A prior history of UTI was noted in 112 patients (13.5%) in the fosfomycin group and 231 patients (11.1%) in the amikacin group. No statistically significant differences were observed between the two groups with respect to these baseline variables. Baseline demographic and clinical characteristics of the study population are summarized in Table 1.

**Table 1:** Baseline demographic and clinical characteristics of the study population PUC: Per-urethral catheter; UTI: Urinary tract infection.

Variables	Amikacin (n = 2083)	Fosfomycin (n = 831)	Statistical Test (Value)	p-Value
Male sex	1641 (78.8%)	588 (70.8%)	𝜒^2 ^= 3.06	0.08
Mean age (years)	51 ± 14	47 ± 12	t = 1.86	0.06
Diabetes mellitus	214 (10.3%)	97 (11.7%)	𝜒^2 ^= 0.98	0.32
PUC present	88 (4.2%)	39 (4.7%)	𝜒^2 ^= 0.30	0.58
Prior UTI	231 (11.1%)	112 (13.5%)	𝜒^2 ^= 2.88	0.09

Indications for cystoscopic procedures

The indications for outpatient CPE were similar in both groups. The most common indications included surveillance cystoscopy for non-muscle-invasive bladder cancer, evaluation of hematuria, assessment of lower urinary tract symptoms, urethral evaluation, and double-J stent removal following endourological procedures such as PCNL, URSL, and pyeloplasty. Post-procedural UTI rates and ADRs are presented in Table 2.

**Table 2:** Post-procedural urinary tract infection rates and adverse drug reactions UTI: Urinary tract infection; ADR: Adverse drug reaction.

Outcome	Amikacin (n = 2083)	Fosfomycin (n = 831)	Statistical Test	p-Value
Symptomatic UTI	139 (6.7%)	48 (8.2%)	𝜒^2 ^(Yates) = 0.65	0.42
Culture-positive UTI	44 (2.1%)	17 (2.0%)	𝜒^2 ^(Yates) = 0.0	0.91
Acute kidney injury	54 (2.6%)	1 (0.1%)	Fisher’s exact	<0.001
Gastrointestinal ADRs	49 (2.3%)	69 (8.3%)	𝜒^2 ^= 52.62	<0.001
Injection-related ADRs	40 (1.9%)	Not applicable	Fisher’s exact	<0.001

Post-procedural urinary tract infection

Symptomatic UTI within 14 days following CPE was observed in 48 patients (8.2%) in the fosfomycin group and 139 patients (6.7%) in the amikacin group. Clinical features included dysuria, urinary frequency or urgency, suprapubic discomfort, and fever requiring antibiotic therapy. The difference in symptomatic UTI rates between the two groups was not statistically significant.

Culture-positive UTI

Among symptomatic patients, culture-positive UTI was documented in 17 patients (2.0%) in the fosfomycin group and 44 patients (2.1%) in the amikacin group. The majority of isolates were Gram-negative organisms, predominantly *Escherichia coli *and *Klebsiella *species. Extended-spectrum beta-lactamase-producing organisms were identified in a subset of cases in both groups. No statistically significant difference was observed in culture-positive UTI rates between the two prophylactic regimens.

ADRs

Renal Adverse Effects

Acute kidney injury (AKI) was observed in one patient (0.1%) in the fosfomycin group and 54 patients (2.6%) in the amikacin group. AKI was defined as an increase in serum creatinine from baseline to ≥0.3 mg/dL during the follow-up period. The incidence of AKI was significantly higher in the amikacin group.

Gastrointestinal Adverse Effects

Gastrointestinal adverse effects were more frequently reported in the fosfomycin group, occurring in 69 patients (8.3%), and included diarrhea, nausea, abdominal discomfort, and vomiting. In comparison, 49 patients (2.3%) in the amikacin group reported gastrointestinal symptoms.

Injection-Related Reactions

Injection-related adverse effects were documented exclusively in the amikacin group. A total of 40 patients (1.9%) experienced injection-site pain, local induration, or phlebitis following intravenous administration.

Allergic Reactions, Ototoxicity, and Serious Adverse Events

Allergic reactions were uncommon in both groups. Fourteen patients in the fosfomycin group and six patients in the amikacin group developed mild allergic manifestations such as rash or pruritus. The number of allergic reactions was small in both groups, and no meaningful statistical comparison was performed. No clinically evident ototoxicity was observed in either group. No serious adverse events, including life-threatening reactions or hospital admissions directly attributable to the prophylactic antibiotic, were recorded.

Summary of Results

Both amikacin and fosfomycin were associated with low rates of post-procedural symptomatic and culture-positive UTI following outpatient CPE. While infection outcomes were comparable, the ADR profiles differed significantly, with amikacin showing higher renal and injection-related toxicity and fosfomycin being associated with a higher incidence of mild gastrointestinal adverse effects.

## Discussion

Antibiotic prophylaxis for cystoscopic procedures aims to balance effective prevention of post-procedural UTI with antimicrobial stewardship and patient safety. Although major guidelines recommend a risk-stratified approach and discourage routine prophylaxis for low-risk diagnostic cystoscopy, antibiotic prophylaxis continues to be widely used in real-world practice, particularly in high-volume outpatient settings [1,4,9]. In this large retrospective cohort study comparing amikacin and fosfomycin as prophylactic agents before outpatient cystoscopic procedures, we observed comparable rates of symptomatic and culture-positive UTI between the two regimens, with clear differences in ADR profiles.

In the present study, symptomatic UTI within 14 days of cystoscopy occurred in 8.2% of patients receiving fosfomycin and 6.7% of those receiving amikacin, a difference that was not statistically significant. Similarly, culture-positive UTI rates were low and nearly identical between the two groups. These findings suggest that single-dose oral fosfomycin provides prophylactic efficacy comparable to that of parenteral amikacin in the outpatient cystoscopy setting. This observation is consistent with previous studies evaluating fosfomycin as prophylaxis for urological procedures, which have demonstrated non-inferior infection prevention compared with other commonly used agents [2,5-7].

Baseline characteristics, including diabetes mellitus, presence of a PUC, and prior history of UTI, were comparable between the two groups. This reduces the likelihood that differences in infection outcomes were driven by baseline risk imbalance. The consistency of infection rates across a large and heterogeneous real-world population supports the effectiveness of fosfomycin as a prophylactic option when used in appropriately selected patients undergoing outpatient cystoscopy. Inclusion of patients with asymptomatic bacteriuria reflects real-world practice; however, post-procedural infection was defined strictly by clinical symptoms, minimizing misclassification.

A major strength of this study is its large sample size, which allows meaningful evaluation of both efficacy and safety outcomes. Many earlier studies examining antibiotic prophylaxis for cystoscopy have been limited by small cohorts or selective patient populations [2,8]. In contrast, the present study reflects routine clinical practice in a high-volume tertiary care center, enhancing the generalizability of the findings to everyday outpatient urological practice.

The most striking finding of this study relates to ADRs, particularly renal toxicity. AKI was observed in 2.6% of patients receiving amikacin, compared with only one patient (0.1%) in the fosfomycin group. This significant difference is consistent with the well-recognized nephrotoxic potential of aminoglycosides, which is mediated by proximal tubular accumulation and oxidative injury, and may occur even with short-term or single-dose exposure [13]. Although aminoglycoside-associated nephrotoxicity is often considered dose- and duration-dependent, our findings highlight that clinically relevant renal impairment can still occur in the prophylactic setting.

The occurrence of AKI in one patient receiving fosfomycin emphasizes that no antibiotic is entirely devoid of adverse effects. However, the markedly lower incidence compared with amikacin reinforces the renal safety advantage of fosfomycin, particularly in elderly patients, diabetics, and those with borderline renal function [14]. This is especially relevant in outpatient settings, where routine post-procedure renal monitoring may not be consistently performed.

Gastrointestinal adverse effects were more frequently observed in the fosfomycin group, affecting 8.3% of patients. These included diarrhea, nausea, abdominal discomfort, and vomiting. Importantly, these adverse effects were mild and self-limiting and did not require hospitalization or treatment discontinuation. Similar gastrointestinal tolerability profiles have been reported in previous studies evaluating fosfomycin in urological and non-urological settings [6,14]. In contrast, gastrointestinal symptoms were less frequent in the amikacin group, but this advantage must be weighed against the higher risk of renal toxicity.

Injection-related adverse reactions were observed exclusively in the amikacin group and included injection-site pain, local induration, and phlebitis. Although not life-threatening, these reactions contribute to patient discomfort, increase nursing workload, and reduce the overall convenience of parenteral prophylaxis. Fosfomycin, being orally administered, avoids these issues entirely and offers a practical advantage in busy outpatient and day-care settings [15].

No clinically evident ototoxicity was observed in either group, which is reassuring given concerns regarding aminoglycoside-associated vestibulo-cochlear toxicity. However, the retrospective nature of the study and reliance on symptom-based documentation may underestimate subclinical ototoxicity, which represents an inherent limitation.

From a guideline perspective, both the EAU and the AUA recommend selective antibiotic prophylaxis for cystoscopy based on patient-specific risk factors, rather than routine administration in all patients [1,4]. Although prophylaxis continues to be frequently employed in real-world practice, the findings of the present study support this selective approach and suggest that when prophylaxis is deemed necessary, oral fosfomycin represents an appropriate alternative to aminoglycosides, with comparable efficacy and a more favorable short-term safety profile. However, our findings should not be interpreted as support for routine prophylaxis in low-risk diagnostic cystoscopy but rather inform antibiotic selection when prophylaxis is deemed necessary based on risk stratification.

Cost considerations are also relevant. In our setting, fosfomycin was associated with a higher direct drug cost compared with amikacin. However, this difference should be interpreted in the context of indirect costs related to intravenous administration, consumables, personnel time, and management of adverse events. Although a formal cost-effectiveness analysis was not performed, the reduced nephrotoxicity, avoidance of injection-related complications, and improved convenience associated with fosfomycin may partially offset its higher acquisition cost.

This study has limitations inherent to its retrospective design, including potential selection bias, lack of randomization, and absence of detailed microbiological resistance analysis. The choice of prophylactic antibiotic was based on institutional practice rather than predefined allocation, and unmeasured confounding factors may exist. The absence of a no-prophylaxis comparator group is a limitation of this retrospective design and warrants evaluation in future prospective studies. Nevertheless, the large sample size and real-world nature of the cohort strengthen the validity and applicability of the findings.

Therefore, fosfomycin and amikacin demonstrated comparable efficacy in preventing post-cystoscopy UTI. However, fosfomycin was associated with a significantly more favorable safety profile, particularly with respect to renal toxicity and injection-related adverse effects. These findings support fosfomycin as a reasonable alternative to aminoglycosides when prophylaxis is indicated for antibiotic prophylaxis before outpatient cystoscopic procedures, especially in patients at risk for aminoglycoside-related toxicity. Prospective randomized studies are warranted to further validate these observations and refine patient selection for prophylactic antibiotic use.

## Conclusions

Antibiotic prophylaxis before cystoscopic procedures must balance infection prevention with safety and stewardship. In this large real-world retrospective study, amikacin and fosfomycin showed comparable efficacy in preventing post-procedural symptomatic and culture-positive UTI. However, safety profiles differed meaningfully. Amikacin was associated with higher rates of AKI and injection-related complications, whereas fosfomycin mainly caused mild, self-limiting gastrointestinal symptoms. The oral administration and favorable renal safety of fosfomycin provide practical advantages in outpatient settings. Despite higher acquisition cost, these benefits may offset indirect costs linked to parenteral therapy and adverse events. Overall, fosfomycin represents a safe, effective alternative option.
